# Induction of lipid oxidation by polyunsaturated fatty acids of marine origin in small intestine of mice fed a high-fat diet

**DOI:** 10.1186/1471-2164-10-110

**Published:** 2009-03-16

**Authors:** Evert M van Schothorst, Pavel Flachs, Nicole LW Franssen-van Hal, Ondrej Kuda, Annelies Bunschoten, Jos Molthoff, Carolien Vink, Guido JEJ Hooiveld, Jan Kopecky, Jaap Keijer

**Affiliations:** 1 Food Bioactives group, RIKILT – Institute of Food Safety, Wageningen UR, PO Box 230, 6700 AE Wageningen, the Netherlands; 2 Department of Adipose Tissue Biology, Institute of Physiology, Academy of Sciences of the Czech Republic, Prague, Czech Republic; 3 Nutrition, Metabolism and Genomics Group, Division of Human Nutrition, Wageningen University, Wageningen, the Netherlands; 4 Human and Animal Physiology, Wageningen University, Wageningen, the Netherlands

## Abstract

**Background:**

Dietary polyunsaturated fatty acids (PUFA), in particular the long chain marine fatty acids docosahexaenoic (DHA) and eicosapentaenoic (EPA), are linked to many health benefits in humans and in animal models. Little is known of the molecular response to DHA and EPA of the small intestine, and the potential contribution of this organ to the beneficial effects of these fatty acids. Here, we assessed gene expression changes induced by DHA and EPA in the wildtype C57BL/6J murine small intestine using whole genome microarrays and functionally characterized the most prominent biological process.

**Results:**

The main biological process affected based on gene expression analysis was lipid metabolism. Fatty acid uptake, peroxisomal and mitochondrial beta-oxidation, and omega-oxidation of fatty acids were all increased. Quantitative real time PCR, and -in a second animal experiment- intestinal fatty acid oxidation measurements confirmed significant gene expression differences and showed in a dose-dependent manner significant changes at biological functional level. Furthermore, no major changes in the expression of lipid metabolism genes were observed in the colon.

**Conclusion:**

We show that marine n-3 fatty acids regulate small intestinal gene expression and increase fatty acid oxidation. Since this organ contributes significantly to whole organism energy use, this effect on the small intestine may well contribute to the beneficial physiological effects of marine PUFAs under conditions that will normally lead to development of obesity, insulin resistance and diabetes.

## Background

Diets rich in polyunsaturated fatty acids (PUFA) of n-3 series show many beneficial health effects, both in animal models and humans. These include effects on cardiovascular and immune systems, on glucose homeostasis, as well as on the accumulation of body fat (e.g. reviewed by [1-3]). However, recent epidemiological studies started a debate on the possible health benefits of n-3 PUFA [4,5]. To resolve the potential health benefits of these fatty acids, knowledge of the underlying mechanisms is needed.

To elucidate molecular effects of n-3 PUFA *in vivo*, gene expression analyses have been undertaken in animal models using a variety of dietary fatty acids in several tissues, including brain, liver, heart, and adipose [6-16]. The majority of those studies focused on liver and white adipose tissue (WAT), which is not surprising given the fact that these are considered the main target organs in a dietary intervention with fatty acids. Since the intestine contributes to a significant extend to the resting metabolic rate and daily energy expenditure [17], it is of relevance to also understand the effects on this organ. Recent studies [18,19] also showed a clear and significant difference of intestinal gene expression between diets high in diacylglycerol versus triacylglycerol, indicating a profound contribution of the small intestine to fatty acid metabolism. Moreover, induction of lipid catabolism genes in the intestine may be involved in the anti-obesity effect of diacylglycerols as compared with triacylglycerols [18,19] and it may even contribute to a differential sensitivity of two inbred mice strains to an obesogenic high-fat diet [20].

Since the most prominent health benefits have been associated with the long-chain n-3 PUFA of marine origin (for references see [21,22]), we have investigated the molecular effects of eicosapentaenoic acid (EPA; 20:5 n-3) and docosahexaenoic acid (DHA; 22:6 n-3) in n-3 high-fat diets. These diets, which do not differ in the total amount of fat relative to control, will be further referred to as EPA&DHA. In our previous studies using similar diets, we showed an anti-adipogenic effect of EPA&DHA [8,23], which was associated with induction of mitochondrial biogenesis and beta-oxidation of fatty acids in WAT, but not in the liver [8].

We hypothesized that, using long-term dietary intervention studies, dietary fatty acid composition may modulate gene expression and lipid metabolism in the intestine, and that especially EPA and DHA may stimulate expression of genes involved in lipid catabolism. To examine this, we performed gene expression analysis of the mouse small intestine and colon, using whole genome oligonucleotide arrays and validation experiments using quantitative real time PCR (qRT-PCR). Results were confirmed in an additional animal experiment by both qRT-PCR and functional intestinal fatty acid beta oxidation measurements.

## Results

### Phenotypic effects of EPA&DHA

Dietary intervention with EPA&DHA in wildtype mice resulted in anti-adipogenic and anti-diabetic effects as described before: significantly lower body weight and epididymal adipose tissue weight, while food intake was non-significantly different [8,23]. Furthermore, the intake of EPA&DHA increased adiponectin expression and secretion from WAT, and protected the mice against induction of insulin resistance by the high-fat diet [23]. Indeed, glucose tolerance tests showed significantly increased glucose tolerance (decreased area under the curves) by increasing amounts of EPA&DHA in the diets, correlating with decreased fasting plasma insulin levels (data not shown). This was associated with induction of mitochondrial biogenesis and beta-oxidation of fatty acids in WAT based on gene and protein expression, but not in the liver [8]. However, the role of the intestine in the improved whole-body metabolic phenotype has not yet been analysed in detail.

### Effects of EPA&DHA on small intestinal gene expression; analysis of genes expressed in both control and intervention group

To investigate the effects of EPA&DHA on gene expression in the intestinal tract, we isolated RNA from scrapings from small intestine of mice following a 4-week dietary intervention. We compared, using whole genome microarray analysis as initial step, the control sHF diet, which was rich in ALA and free of EPA or DHA, with the isocaloric sHFf-F2 diet, in which 44% of lipids were replaced by an EPA and DHA concentrate.

In total, 155 probesets, corresponding to 134 well-annotated genes, showed a significant regulation with an absolute fold change (FC) ≥ 2.0 [see Additional file 1]. This list of 134 genes comprised 110 unique genes, and increased expression was observed for 80 (102 probesets) unique genes, while 30 (32 probesets) unique genes showed decreased expression (Table 1). Functional interpretation using gene ontology and pathway analysis showed metabolism as highly regulated, which included changes in fatty acid uptake, fatty acid oxidation and cholesterol biosynthesis, amongst others. Of note, most, if not all, pathways selected were linked together by the energy molecule acetyl-CoA. The approach using a FC ≥ 1.5 as threshold resulted in a similar list of regulated pathways, of which metabolism, and especially lipid metabolism ranked highest (a more detailed description of these approaches and detailed results are described additionally [see Additional file 2]). Since it is known that a dietary intervention at physiological levels generally gives small effects on gene expression changes ([24,25], own unpublished observations), and the sufficiently large group of genes with FC ≥ 2.0, we focussed on those 100 unique, differentially expressed genes (see Table 1).

**Table 1:** Differentially expressed, unique genes classified in biological processes.

Process	Upregulated	Downregulated	Total
Metabolism	40	9	49
Transport	4	3	7
Apoptosis/cell cycle	4	2	6
Cell adhesion/homeostasis/structure	4	1	5
Digestion	4	0	4
Immune system	1	3	4
Hemopoiesis	1	2	3
Transcription/translation	3	0	3
Signal transduction	2	0	2
Steroid hormone metabolism	2	0	2
Miscellaneous/unknown	8	7	15

Total:	73	27	100

Unique genes (expressed in both dietary groups, FC ≥ 2.0) are classified in 11 processes, and ranked on total number of genes per process. Number of genes upregulated or downregulated by EPA&DHA in small intestine is as indicated.

Detailed inspection of the expression data ([see Additional file 1]; see also for full names of the genes) revealed that EPA&DHA induced expression of the genes involved in branched chain and/or straight chain fatty acid β-oxidation occurring in peroxisomes and mitochondria, and fatty acid ω-oxidation, the latter is indicated by the increased expression of *Cyp4a10 *(FC = 5.6). Furthermore, induction of the peroxisomal biogenesis factor gene *Pex11a *(FC = 2.2) might imply an increase in the number of peroxisomes.

In mitochondria, β-oxidation downstream of peroxisomal branched chain oxidation was upregulated by EPA&DHA, as suggested by increased expression of the genes *CPT1a *(FC = 2.9), and *Hadhb *(FC = 2.2) amongst others. Activity of carnitine palmitoyltransferase 1 (encoded by *Cpt1a*) is rate-limiting in mitochondrial fatty acid uptake for β-oxidation. The gene encoding 3-hydroxy-3-methyglutaryl-CoA synthase (*Hmgcs2*) was also very strongly upregulated (FC = 4.6), in accordance with the fact that in liver this enzyme is the rate limiting enzyme in the synthesis of ketone bodies from the acetyl-CoA generated by fatty acid β-oxidation. Importantly, also expression of *Pdk4 *was increased by EPA&DHA (FC = 3.0), which strongly suggested a switch from glycolysis to fatty acid oxidation [26]. Many of the aforementioned genes are targets of peroxisome proliferator activated receptor (*PPAR*) alpha [27,28], which itself was also upregulated by EPA&DHA (FC = 2.0).

Cholesterol uptake from the lumen into intestinal tissue was increased as observed by increased gene expression of *Cd36 *(FC = 2.3) and *Scarb1 *(FC = 2.2) [29]. Simultaneously, of the 21 genes included in the cholesterol biosynthesis pathway from acetyl-CoA to cholesterol, 2 were not expressed, while the majority showed an inhibition by EPA&DHA, including the rate-limiting enzyme squalene epoxidase (*Sqle*, FC = -2.1). Clearly, such a cooperative inhibition of the majority of the genes in this pathway suggests an orchestrated function within the small intestine. However, the main transcription factor regulating this pathway, *Srebf1 *(SREBP), did not show differential expression. Regulation by n-3 PUFAs was likely given the fact that we could not detect a difference in the cholesterol content between the two diets (data not shown), but the exact mechanisms involved remain unexplained. Finally, downstream steroid hormone biosynthesis was upregulated as shown by a few family members of *Hsd3b *(*Hsd3b2*: FC = 2.3; *Hsd3b3 *FC = 2.0) and *Hsd17b *(*Hsd17b4 *two probesets: FC = 1.6 and 2.0; *Hsd17b13 *FC = 2.4).

### Transcription factor identification by promoter analysis

Literature data and annotation analysis was used to filter the set of regulated genes (see Methods). From the resulting 50 unique genes the promoter region was retrieved, which was used for enrichment analysis of transcription factor binding sites (TFBS). This resulted in a network of 47 genes and linked transcription factors (TFs) that comprised 42 unique input genes, including two TFs (PPARalpha and Dbp), and another 5 non-regulated TFs (NF-κB, Stat3, Sp1, Ahr, and Arnt1; [see Additional file 3]). The majority of differentially regulated genes contained binding sites for two transcription factors: PPARalpha (27 genes), which itself was significantly differentially regulated (see above), and NF-κB (20 genes). These data are in line with the well-known capacity of fatty acids to activate PPARalpha [30], as well as their known effects on NF-κB and Stat3 [3,31].

### Effects of EPA&DHA on small intestinal gene expression; analysis of genes expressed exclusively in either control or intervention group

In addition to the genes that showed expression above the threshold in both groups, we also analysed the 159 probesets being expressed in only one diet group and showing differential expression. We focused on those genes showing a FC ≥ 2.0. In this way, fidelity of the dataset is within acceptable limits as most of these genes are expressed at low abundance. This resulted in 23 unique genes expressed only in the mice fed the EPA&DHA diet, while 47 unique genes showed only expression in the control group. All genes were classified into biological processes (Table 2, [see Additional file 4]), which largely overlapped with the pathways identified using the first subset. Of note, metabolism was also ranked highest, among which are the lipid metabolism genes acetyl-Coenzyme A carboxylase-β (*Acacb*), *Cpa2*, and the peroxisomal protein encoded by *Acot3*, which all showed an induction from being absent in the control group to clear expression in EPA&DHA dietary group.

**Table 2:** Differentially expressed genes with exclusive expression in only EPA&DHA or control dietary group.

Process	Expressed in EPA & DHA diet	Expressed in control diet	Total
Metabolism	11	4	15
Transcription/translation/splicing	3	6	9
Cell adhesion/proliferation/differentiation/structure	-	7	7
Signal transduction	-	7	7
Transport	1	5	6
Protein folding/modification	1	4	5
Cell cycle	1	1	2
Growth factor	1	1	2
Miscellaneous/unknown	5	12	17

Total:	23	47	70

Annotated, unique genes (FC ≥ 2) were classified in biological processes. Processes were ranked on total number of genes being differentially expressed in small intestine.

### Inter-individual variation analysis and confirmation by real time qRT-PCR

Confirmation of differentially expressed genes was first performed using qRT-PCR. We selected a large panel of target genes covering multiple pathways and absolute FC values ranging from 1.3 to > 4. Expression changes (up- or downregulation) were confirmed for all genes (Table 3) and the FC was quite similar in all cases when compared to the microarray data.

**Table 3:** Microarray data validation by qRT-PCR

		Microarray ratio	qRT-PCR ratio	
			
Genes	Probeset ID	Pool	Pool	mean ratio (n = 10–11)
Acaa1a	1456011_x_at	1.67	2.00	2.52 *
Acaa1b	1424451_at	2.19	1.75	
Acaa1a/Acaa1b	1416946_a_at	1.87		
	1416947_s_at	1.59		
Acacb	1427052_at	6.9 ^a^	n.d.^c^	3.91 *
Acox1	1416409_at	1.24	1.34	
	1416408_at	1.35		
Acox2	1420673_a_at	2.91	1.97	
Acox3	1420684_at	n.c.^b^	-1.23	
Cpt1a	1434866_x_at	2.87	1.87	2.79 **
	1438156_x_at	2.60		
Cpt2	1416772_at	1.39	1.44	
Ela2	1448281_a_at	2.04	4.26	
Ela3b	1415884_at	2,64	7.89	
	1435611_x_at	4,20		
	1415883_a_at	4,38		
	1437326_x_at	4,72		
	1435012_x_at	5,17		
H2-Q10	1425137_a_at	-8.22	-2.39	
Hmgcs1	1433443_a_at	-1.82	-2.00	
	1433446_at	-1.57		
Hmgcs2	1431833_a_at	3.61	3.97	
	1423858_a_at	4.56		
Hsd3b2/6	1460232_s_at	2.50	n.d.	1.91 *
Hsd3b3	1427377_x_at	2.00		
Mod1	1416632_at	2.16	2.60	
	1430307_a_at	2.13		
Pck1	1423439_at	2.17	2.21	
	1439617_s_at	2.46		
Pdk4	1417273_at	3.01	n.d.	3.24 *
Sqle	1415993_at	-2.14	-2.35	-1.55 *

Genes are sorted alphabetically. In several cases gene family members were included for validation, although they did not show differential microarray gene expression. Data is represented by ratio of EPA&DHA/control diet. For qRT-PCR, data shown is obtained using a pool of samples (pool), and/or individual samples (EPA/DHA: n = 11, control diet: n = 10) and the mean ratio was calculated. ^a ^this probeset is non-expressed (absent) in control diet; ratio is 6.9; ^b ^n.c.: not changed. ^c ^n.d.: not analyzed; statistical significance was calculated using Student's *t*-test: * p < 0.05, ** p < 0.01.

**Figure 1:**
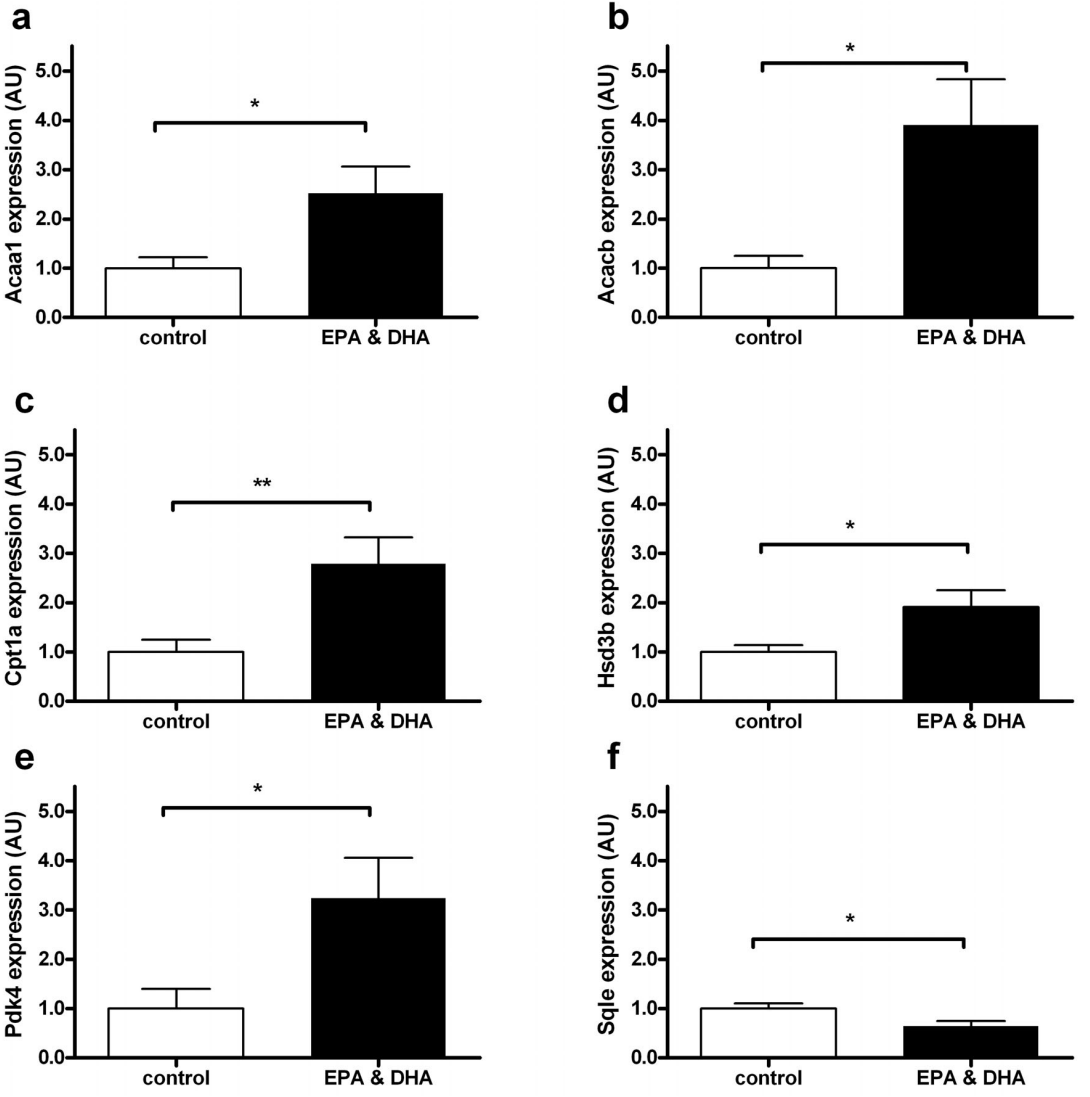
**Gene expression in small intestine**. Quantatative real-time PCR (qRT-PCR) was used to determine normalized gene expression levels in small intestine of individual wildtype mice that received the control diet (n = 10, white bars) or EPA&DHA diet (n = 11, black bars). Gene expression levels were normalized using calnexin and averaged per group; the mean expression level of the control group was arbitrarily set at 1. Bars are presented as mean ± standard error. Genes shown (with the mean ratio between the groups shown in parenthesis) are **a **Acaa1 (2.52), **b **Acacb (3.91), **c **Cpt1a (2.79), **d **Hsd3b (1.91), **e **Pdk4 (3.24), **f **Sqle (-1.55). Statistical significance was analyzed using Student's *t*-test: * p < 0.05, ** p < 0.01. AU, arbitrary units.

To investigate in more detail inter-individual variation in gene expression and significance of the induced gene expression changes by nutritional treatment, we selected the following 6 genes representing the major pathways being influenced by the diet intervention (see above): fatty acid β-oxidation in peroxisomes (*Acaa1a*) and mitochondria (*Cpt1a *and *Acacb*), the switch between glycolysis and fatty acid oxidation (*Pdk4*), biosynthesis of steroid hormones (*Hsd3b*), and biosynthesis of cholesterol (*Sqle*). For all genes we observed that the mean gene expression ratios (n = 10–11 per dietary group) were similar to the observed ratios by microarray analysis of pooled samples. More importantly, gene expression changes using individual samples were statistically significant for all genes (Figure 1, Table 3).

### Colon

**Figure 2:**
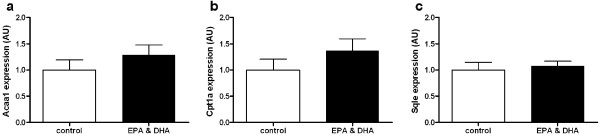
**Gene expression in colon**. qRT-PCR was used to determine normalized gene expression levels in colon of individual wildtype mice that received the control diet (n = 12, white bars) or EPA&DHA diet (n = 11, black bars). Gene expression levels were normalized using calnexin and averaged per group; the mean expression level of the control group was arbitrarily set at 1. Bars are presented as mean ± standard error. Genes and fold changes in parenthesis shown are **a **Acaa1 (1.29), **b **Cpt1a (1.37), **c **Sqle (1.08). All changes are non-significant (p > 0.2, Student's *t*-test). AU, arbitrary units.

The genes *Acaa1a*, *Cpt1a*, and *Sqle*, representing three pathways differentially regulated in small intestine, were analyzed by qRT-PCR in individual colon samples (n = 10). In none of the cases a significant difference in gene expression was observed (Figure 2). Exclusion of the two animals from the control group which showed lowest expression for these three genes (10-fold or more) did not influence the outcome of this analysis (maximum FC < 1.15, all non-significant; data not shown). This underscores the different function of the small intestine (mainly uptake and transfer of nutrients) and the colon (mainly water absorption).

### Biological and physiological validation in 2^nd ^animal experiment

**Figure 3:**
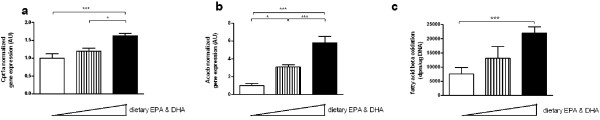
**Dose-dependent intestinal gene expression and fatty acid beta oxidation**. In a second animal experiment, intestinal gene expression analysis was used to confirm and biologically validate observations in a dose-dependent manner. Gene expression analysis was performed using qRT-PCR of small intestinal samples of individual wildtype mice that received the control diet (n = 9, white bars), EPA&DHA diet (n = 9, black bars), and intermediate levels of dietary EPA&DHA (n = 9, hatched bars). The triangle shows increasing dietary EPA&DHA content. Gene expression levels were normalized using calnexin and averaged per group; the mean expression level of the control group was arbitrarily set at 1. Bars represent mean ± standard error. Genes shown are **a: **Cpt1a, and **b: **Acacb. Functional fatty acid beta oxidation was used for biological validation (**c**). Intestinal tissue fatty acid beta oxidation (n = 7–9) is shown for mice that received control diet (white bars), EPA&DHA diet (black bars), and intermediate levels of dietary EPA-DHA (hatched bars). The triangle shows increasing dietary EPA&DHA content. Statistical significance was analyzed using one-way ANOVA and Tuckey's post hoc tests: * p < 0.05, *** p < 0.01. AU, arbitrary units.

To further validate the observed significant gene expression changes, we performed an independent second animal experiment and analysed small intestinal gene expression by qRT-PCR. Moreover, the increased gene expression suggesting increased intestinal fatty acid beta oxidation was functionally analysed, and finally, we used two EPA&DHA diets in order to investigate a dose-response relationship. In this experiment, cHF-based diets were used. Although the micronutrient composition of the control cHF diet is not defined as precisely as the semisynthetic sHFf diet used in the first experiment, and it is based on (n-6 PUFA rich) corn oil rather than on (ALA-rich) flaxseed, the control cHF diet promotes accumulation of fat much better compared with the sHFf diet and hence it allows for the detection of the anti-obesity effect of n-3 PUFA [22]. In addition to the control cHF diet, also two other cHF-based diets with 15 and 44% of its lipids replaced by EPA&DHA (cHF-F1 and cHF-F2, respectively) were used. Gene expression analysis of genes involved in mitochondrial beta oxidation (*Cpt1a *and *Acacb*) independently and significantly confirmed the observed significant differences (Figure 3a,b). Moreover, these results showed a significant dose-response effect: increased gene expression upon increased EPA&DHA content in the diets (Figure 3a,b). Furthermore, functional analysis indeed showed a significant dose-dependent increase in intestinal fatty acid beta oxidation upon increased EPA&DHA content in the diet compared to control diet: lowest levels in the control group, intermediate increased levels in the cHF-F1 group and highest levels in the cHF-F2 group (Figure 3c).

## Discussion

Dietary intake of EPA&DHA induced changes in gene expression in the small intestine, including many metabolic genes. Especially genes involved in lipid catabolism were upregulated. In contrast, a large cluster of the genes engaged in cholesterol biosynthesis was downregulated. Importantly, all these effects were specifically induced by long-chain n-3 PUFA, EPA and DHA, as compared with their precursor ALA, and could not be detected in the colon for a selected set of genes.

It is tempting to suggest that increased catabolism of lipids induced by EPA&DHA in the small intestine contributes to the complex and beneficial effects of n-3 PUFA of marine origin. The small intestine mediates the entry of nutritional lipids and is one of the main sites of β-oxidation [32-34]. Therefore, an increase in lipid oxidation in the intestine may exert a hypolipidemic effect, i.e. one of the most pronounced effects of EPA and DHA in mammals (reviewed in [1,3]). This effect of EPA&DHA in the intestine is surprisingly similar to the enhanced lipid oxidation induced by diacylglycerols versus triacylglycerols [18,19]. These two types of treatments both have relatively little effects in liver and muscle ([20]; own unpublished data). In contrast, in white adipose tissue, intake of EPA&DHA induced genes of fatty acid oxidation, as well as quite specifically, mitochondrial biogenesis [8]. Taken together, EPA and DHA orchestrate gene expression adaptations in many tissues, including the intestine.

Recent studies addressing the gene expression changes of intestinal tissue upon fish oil or fatty acids, focussed on barrier genes only [16] or a focussed limited number of genes by qRT-PCR [34], while here a whole genome approach was used. This allows for detection of changes not only in the most likely pathways, but also in pathways not foreseen. Our study supports the findings by Mori et al. [34] for the majority of their selected genes analyzed (*Cpt1a, Mod1, Pdk4, Hmgcs2, Cyp4a10*, and *Acadm*), as well as for barrier gene expression ([16], data not shown). Unexpectedly, we observed intestinal downregulation of cholesterol biosynthesis due to EPA&DHA, although this is in agreement with a similar effect observed in murine livers after tuna fish oil feeding [11]. In addition, this might coincide with a possible increase in cholesterol absorption as observed from *ScarB1*, *Cd36*, *Abca1 *and *Ela3B *gene expression, even with identical cholesterol content of the diets. Intracellular homeostasis may decrease cholesterol biosynthesis to counteract increased influx. Maintenance of homeostasis is supported by non differential expression of *Soat2*/ACAT, involved in cholesterol esterification and of other genes in cholesterol metabolism (HMG-CoA reductase, *Npc1l1*, *Mttp, Abcg5, Abcg8, Nr1h2 *(*LXRβ*) and *Nr1h3 *(*LXRα*)). The intestinal lack of regulation of *Srebf1*/SREBP further strengthen the observations that PUFA regulation of SREBP that accounts for PUFA-mediated suppression of gene expression seems to be liver-specific [13]. Furthermore, of the regulatory machine known to be induced by DHA and EPA (PPARs, LXRs, HNF4A, and SREBPs), only PPARα showed differential expression in murine small intestine. This is further supported by our promoter-analysis of the differentially regulated genes, which showed PPARα as the major transcription factor involved.

Moreover, most if not all tissues analysed thus far show an increased energy metabolism upon n-3 FFA, and our results support the notion of the beneficial effects of fish oils independent of its n-3 effect.

Genes engaged in lipid oxidation and ketogenesis are in general upregulated in small intestine [35], liver [20], and skeletal muscle [36] by an increase in dietary fat content. When activated in the muscle, ketogenesis marks a metabolic disconnection between β-oxidation and tricarboxylic acid cycle and could lead to insulin resistance [36]. In our study however, we compared diets with equal fat content in control and intervention groups, which only differed in their fatty acid composition. This implies that marine PUFAs specifically induce lipid catabolism in intestine. Furthermore, in comparison with another high fat Western diet [35], we observed similar (up or down) gene expression regulation by EPA&DHA diet (e.g. *Angplt4*, *Gsn*, and *Smpdl3*), as well as an inverse regulation (e.g. *ApoC2 *and *H2Q10 *are upregulated by a high-fat diet [35], but downregulated by EPA&DHA). Differences might be explained by the fatty acid content in the diets used.

Despite decreased adiposity due to EPA&DHA in the diet, these fatty acids have not affected total energy intake [8,22], and also content of lipids in faeces was unaffected [22]. This strongly suggests a higher energy expenditure in the animals exposed to EPA&DHA. The results presented here indicate that one of the organs that physiologically contribute to increased oxidation of fatty acids is the small intestine.

## Conclusion

In conclusion, we present data showing the involvement of small intestine in the complex changes of lipid metabolism exerted by long term dietary intake of EPA and DHA by gene expression analysis and functional *ex-vivo *beta oxidation analysis. Furthermore, we show that these effects are regulated in a dose-dependent manner. In view of its large contribution to overall energy metabolism, modulation of gene expression and metabolism in the intestine by dietary lipids, and especially long-chain n-3 PUFA of marine origin, represents a promising target for the prevention of obesity and associated co-morbidities.

## Methods

### Animals and diets

In the first experiment, male 4-month-old C57BL/6J mice were maintained for 4 weeks on semisynthetic high-fat (20% wt/wt) diets differing in the composition of n-3 PUFA. These mice were already used in our previous study [8]. Two isocaloric diets [8,22] were used (n = 12): control sHFf diet which contained flax-seed oil (rich in ALA) as the only lipid source, or the sHFf-F2 diet, which had the same composition except that 44% of the lipids were replaced by a n-3 PUFA concentrate containing 6% EPA and 51% DHA (EPAX 1050TG; EPAX AS, Lysaker, Norway). This diet is denoted as EPA&DHA throughout the study. At the end of the experiment, mice were killed by cervical dislocation and small intestine from 3 cm under the stomach to caecum was isolated and cut lengthwise. The intestine was washed in 154 mM KCl and scraped. The epithelial cells were collected, frozen in liquid nitrogen and stored at -80°C. This procedure was also performed for 5 cm of colon.

In the second experiment, subgroups (n = 9) of male 4-month-old C57BL/6J mice were fed either (i) control obesity-promoting high-fat (35% lipids wt/wt; cHF) diet derived from standard chow diet based on corn oil, or (ii) and (iii) cHF diet with 15 and 44% of lipids, respectively, replaced by EPAX 1050TG (cHF-F1 and cHF-F2, respectively; see [22]). After 6 weeks, intestinal tissue was isolated for fatty acid beta oxidation and gene expression analysis by quantitative RT-PCR (qRT-PCR) only. Macronutrient composition, energy density, and fatty acid composition of dietary lipids of all the diets used in this study is well characterised [22]. The experiments were conducted under the guidelines for the use and care of laboratory animals of the Institute of Physiology, Academy of Sciences of the Czech Republic.

### RNA isolation

Extraction of total RNA was performed with the use of TRIzol (Invitrogen, Breda, The Netherlands). RNeasy columns (Qiagen, Venlo, The Netherlands) were used to purify the RNA. Quantitative and qualitative measures were performed using Nanodrop ND-1000 Spectrophotometer (NanoDrop Technologies, Delaware, USA). For the first animal experiment, four samples did not pass quality thresholds and were excluded from further analyses (two control small intestine samples and one EPA&DHA small intestine sample and one EPA&DHA colon sample). The remaining RNA samples were pooled per tissue per diet and integrity was analyzed after keeping the samples for 1 hour at 37°C using an Agilent bioanalyzer (Agilent Technologies, Amstelveen, The Netherlands) with a RNA 6000 Nano LabChip kit, according to the manufacturer's instructions. The two pooled colon samples were not used further due to their apparent partial degradation (RNA Integrity Number (RIN) < 7.0). All individual RNA samples from the second animal experiment passed quality control criteria, and were used for subsequent qRT-PCR analysis.

### Array hybridization and scanning

The pools of small intestine RNA samples (control (n = 10) and EPA&DHA (n = 11)) were hybridized on separate Affymetrix MOE430_2 GeneChip mouse arrays (Santa Clara, CA, USA). This array contains 45,102 probesets, detecting over 39,000 transcripts that represent 16,579 unique genes. Detailed methods for labelling and subsequent hybridizations to the arrays are described in the eukaryotic section in the GeneChip Expression Analysis Technical Manual Rev. 3 from Affymetrix, and are available upon request. Arrays were scanned on a GeneChip Scanner 3000 (Affymetrix).

### Microarray data analysis

Quality of the data was assessed on diagnostic plots generated from the raw, non-processed data, as described [37]. All arrays passed these strict criteria and were included in the analyses.

The Affymetrix default algorithm (MAS 5.0) was used to summarize data and significance of observed gene expression changes (present/absent call per probeset per array, fold change (FC) between normalized arrays, and its significance by the p-value). In total, 24270 probesets (54%) showed expression at least in either one of the arrays. Significant differentially expressed probesets were identified by direct comparison between the two dietary groups for all probesets called present on both arrays. Probesets that satisfied the general Affymetrix criterion of t-test probability < 0.27% (p-value < 0.0027) were considered to be significantly regulated, and these were further investigated. Probesets were annotated using information provided by Affymetrix (release of July 12th, 2006) and all gene symbols are presented throughout the article according to Mouse Genome Informatics [38].

Pathway analysis, including ranking, was done using Metacore (GeneGo, St. Joseph, MI, USA). The data of functionally annotated genes only were analyzed in two subsets. In order to successfully rank the most prominent differentially regulated pathway(s), a large set of genes was analyzed. In this way, those pathways showing a high number of significant regulated genes, given the (high) number of genes expressed within the pathway, will rank highest. The first subset comprised those genes of which expression was present in both dietary conditions. Pathway analysis was done using a cut-off of absolute FC ≥ 1.5 or ≥ 2.0 and the p-value per gene as provided by MAS5.0, in order to increase detection of biological relevant regulated pathways. The second subset comprised probesets expressed in only one dietary group, and therefore absent in the other (FC ≥ 2.0). The given FCs as generated by MAS 5.0 were used, although in view of the absence of expression under one dietary condition these may not be reliable, hence the choice of separate analysis. Array data have been submitted to the Gene Expression Omnibus, accession number GSE11936.

### Promoter analysis to identify transcription factor regulation

All differentially regulated probesets, as shown in Table 1, were analyzed using Genomatix BiblioSphere Pathway Edition software, without any pre-selection in up- or down-regulation. Unique genes were identified and those genes plus gene-gene interactions were filtered [39] for organ-specificity using MeSH [Medical Subject Heading] "Digestive System" [A03]. This resulted in a set of 50 unique input genes. Promoter regions of around 650 bp upstream of transcription start site per gene were analysed for known transcription factor binding sites (TFBS) combined with prior knowledge in literature. Criteria for this analysis were: only direct interactions were considered, a minimum of 3 published articles should describe the functional interaction (so called "function word level B2"), and for inclusion of a transcription factor (TF) showing TF-gene interaction, the additional criterion of at least two different input genes having this specific TFBS was compulsory. In this way we analyzed specifically only TF-gene interactions relevant for the gastrointestinal tract.

### Quantitative real time PCR

qRT-PCR was performed according to Van Schothorst et al. [40]. Briefly, 1 μg of total RNA was used for cDNA synthesis. Primers were designed using Beacon Designer (Biosoft International, Palo Alto, USA) and ordered by Biolegio (Malden, The Netherlands). Gene symbols, names, accession numbers and primer sequences are listed in an additional file [see Additional file 5]. Analysis of the reaction efficiency [41] was performed with a dilution series of pooled cDNAs serving as standard curve. Briefly, after a 3 minute denaturation at 94°C, 40 cycles of 15 seconds at 94°C and 30 seconds at 59°C, were followed by a melting curve gradient. Calnexin (*Canx*) and hypoxanthine guanine phosphoribosyl transferase 1 (*Hprt1*) were used as reference genes based on least variation observed in microarray data analysis. *Canx *showed most stable expression and was used and shown in all cases. Endorsable data were obtained using *Hprt1*. C_t_-values of the two pools of dietary groups were measured for 17 genes in triplo and averaged, followed by calculation of the relative gene expression using the 2^-ΔΔCT ^method [42].

Relative target gene expression was measured in more detail using individual samples for the genes *Cpt1a, Acaa1a, Sqle, Acacb, Pdk4 *and *Hsd3b*, of which the latter three were not analysed using pooled RNA samples. Samples were run in duplicate, averaged, and relative gene expression was calculated using the standard curve method. qRT-PCR analyses for the second animal experiment were performed using individual RNA samples for target genes *Cpt1a *and *Acacb*, as described above.

All values are presented as mean ± SE. In case of only 2 groups (first experiment), differences between groups were analyzed using Student t-tests with two-tailed, unequal variances and significance expressed at p < 0.05, or lower as indicated, while differences between three groups (second experiment) were analysed using one-way ANOVA and Tuckey's posthoc tests and significance expressed at p < 0.05, or lower as indicated.

### Fatty acid beta oxidation

Intestinal fatty acid beta oxidation was measured as published [43]. Briefly, samples were incubated in Krebs-Ringer bicarbonate buffer with [^14^C(U)]-palmitate, ^14^CO_2 _was trapped in hyamine hydroxide, quantified by liquid scintillation counting, and oxidation rate was normalized to tissue DNA content [44]. All data are presented as mean ± SE. Differences between groups were analyzed using one-way ANOVA and Tuckey's post hoc tests and considered statistically significance at p < 0.05.

## Authors' contributions

EvS, PF, NFvH, JKo and JKe conceived the studies, supervised the execution and drafted the mansucript. EvS, PF, OK, AB, JM, CV, and GH performed animal studies, RNA isolation, qRT-PCRs, microarray hybridisation and analyses, or fatty acid beta oxidation measurements. All authors have read and approved the final manuscript.

## Supplementary Material

Additional file 1 **Differentially regulated probesets expressed in both dietary groups.** The data provided represent the statistical significant differentially expressed probesets of the microarrays which are shared between the two dietary groups (Fold change ≥ 2.0). Click here for file

Additional file 2 **Additional data analysis of gene expression differences.** Additional and extended microarray data analysis results are described in more detail. Click here for file

Additional file 3 **Transcription factors enriched for involvement with differentially expressed genes.** The data provided represent identified transcription factors on the basis of an enriched presence of TFBS in the promoter region of the set of significantly differentially regulated genes, combined with data from scientific publications. Click here for file

Additional file 4 **Differentially regulated probesets expressed in only one dietary group.** The data provided represent all differentially expressed probesets of the microarrays which are unique for either one of the dietary groups. Click here for file

Additional file 5 **Real-time quantitative PCR: genes and primers.** Gene Symbols, Names, Annotation (RefSeq), and primer sequences for genes analyzed using qRT-PCR are shown. Click here for file
