# Supplementary material for: Induction of lipid oxidation by polyunsaturated fatty acids of marine origin in small intestine of mice fed a high-fat diet
Source: BMC Genomics. 2009 Mar 16;10:110. doi: 10.1186/1471-2164-10-110 (PMC2662879; doi:10.1186/1471-2164-10-110)
Supplement: Additional file 1 — Differentially regulated probesets expressed in both dietary groups. The data provided represent the statistical significant differentially expressed probesets of the microarrays which are shared between the two dietary groups (Fold change ≥ 2.0). [file 1471-2164-10-110-S1.pdf]

Additional file 1. Differentially regulated probesets expressed in both dietary groups.

Process / Affymetrix ID	Symbol	Gene name	Fold Change	I/D
**Metabolism -** upregulated				
1424853_s_at	Cyp4a10	cytochrome P450, family 4, subfamily a, polypeptide 10	6.96	I
1416139_at	Reg2	regenerating islet-derived 2	5.86	I
1435370_a_at	Ces3	carboxylesterase 3	5.54	I
1435012_x_at	Ela3b	elastase 3B, pancreatic	5.17	I
1449065_at	Acot1 = Cte1	cytosolic acyl-CoA thioesterase 1	5.13	I
1437326_x_at	Ela3b	elastase 3B, pancreatic	4.72	I
1423858_a_at	Hmgcs2	3-hydroxy-3-methylglutaryl-Coenzyme A synthase 2	4.56	I
1421868_a_at	Pnlip	pancreatic lipase	4.44	I
1415883_a_at	Ela3b	elastase 3B, pancreatic	4.38	I
1435611_x_at	Ela3b	elastase 3B, pancreatic	4.20	I
1417257_at	Cel	carboxyl ester lipase	3.97	I
1422997_s_at	Acot1 /// Acot2	acyl-CoA thioesterase 1 /// acyl-CoA thioesterase 2	3.89	I
1423436_at	Gsta3	glutathione S-transferase, alpha 3	3.84	I
1431833_a_at	Hmgcs2	3-hydroxy-3-methylglutaryl-Coenzyme A synthase 2	3.61	I
1433431_at	Pnlip	pancreatic lipase	3.48	I
1449081_at	Ces3	carboxylesterase 3	3.34	I
1449486_at	Ces1	carboxylesterase 1	3.25	I
1417273_at	Pdk4	pyruvate dehydrogenase kinase, isoenzyme 4	3.01	I
1422716_a_at	Acp1	acid phosphatase 1, soluble	2.95	I
1420673_a_at	Acox2	acyl-Coenzyme A oxidase 2, branched chain	2.91	I
1434866_x_at	Cpt1a	carnitine palmitoyltransferase 1a, liver	2.87	I
1415777_at	Pnliprp1	pancreatic lipase related protein 1	2.85	I
1427912_at	Cbr3	carbonyl reductase 3	2.75	I
1421040_a_at	Gsta2	glutathione S-transferase, alpha 2 (Yc2)	2.73	I
1419104_at	Abhd6	abhydrolase domain containing 6	2.64	I
1415884_at	Ela3b	elastase 3B, pancreatic	2.64	I
1438156_x_at	Cpt1a	carnitine palmitoyltransferase 1a, liver	2.60	I
1438310_at	Dgkh	Diacylglycerol kinase, eta	2.53	I
1450884_at	Cd36	CD36 antigen	2.50	I
1419395_at	Acot12 = Cach	cytosolic acetyl-CoA hydrolase	2.46	I
1439617_s_at	Pck1	phosphoenolpyruvate carboxykinase 1, cytosolic	2.46	I
1460674_at	Mpra = Paqr7	membrane progestin receptor alpha	2.41	I
1460606_at	Hsd17b13	hydroxysteroid (17-beta) dehydrogenase 13	2.39	I
1421011_at	Dhrs8	dehydrogenase/reductase (SDR family) member 8	2.38	I
1419365_at	Pex11a	peroxisomal biogenesis factor 11a	2.36	I
1455820_x_at	Scarb1	scavenger receptor class B, member 1	2.25	I
1422438_at	Ephx1	epoxide hydrolase 1, microsomal	2.22	I
1416050_a_at	Scarb1	scavenger receptor class B, member 1	2.22	I
1424451_at	Acaa1	3-ketoacyl-CoA thiolase B	2.19	I
1423166_at	Cd36	CD36 antigen	2.19	I
1423437_at	Gsta3	glutathione S-transferase, alpha 3	2.19	I
1437172_x_at	Hadhb	hydroxyacyl-Coenzyme A dehydrogenase/3-ketoacyl-Coenzyme A thiolase/enoyl-Coenzyme A hydratase (trifunctional protein), beta subunit	2.19	I
1422780_at	Pxmp4	peroxisomal membrane protein 4	2.19	I
1450883_a_at	Cd36	CD36 antigen	2.17	I
1423439_at	Pck1	phosphoenolpyruvate carboxykinase 1, cytosolic	2.17	I
1416632_at	Mod1	malic enzyme, supernatant	2.16	I
1439428_x_at	Gmds	GDP-mannose 4, 6-dehydratase	2.14	I
1450391_a_at	Mgll	monoglyceride lipase	2.13	I
1430307_a_at	Mod1	malic enzyme, supernatant	2.13	I
1449442_at	Pex11a	peroxisomal biogenesis factor 11a	2.08	I
1437378_x_at	Scarb1	scavenger receptor class B, member 1	2.08	I
1416429_a_at	Cat	catalase	2.07	I
1419510_at	Es22	esterase 22	2.06	I
1441860_x_at	Ide	Insulin degrading enzyme	2.01	I

1419622_at	Ugt2b5	UDP-glucuronosyltransferase 2 family, member 5	2.01	I
1436298_x_at	Paics	phosphoribosylaminoimidazole carboxylase, phosphoribosylaminoribosylaminoimidazole, succinocarboxamide synthetase	2.00	I
1450037_at	Usp9x	ubiquitin specific protease 9, X chromosome	2.03	MI
**Metabolism - downregulated**				
1436712_at	Pla2g4c	phospholipase A2, group IVC (cytosolic, calcium-independent)	9.78	D
1444662_at	Pla2g4c	phospholipase A2, group IVC (cytosolic, calcium-independent)	5.66	D
1449907_at	Bcmo1	beta-carotene 15,15'-monooxygenase	2.20	D
1423418_at	Fdps	farnesyl diphosphate synthetase	2.20	D
1415993_at	Sqle	squalene epoxidase	2.14	D
1450788_at	Saa1	serum amyloid A 1	2.10	D
1428140_at	Oxct1	3-oxoacid CoA transferase 1	2.04	D
1450646_at	Cyp51	Cytochrome P450, family 51	2.03	D
1421821_at	Ldlr	Low density lipoprotein receptor	2.01	D
1419075_s_at	Saa1 / Saa2	serum amyloid A 1 / serum amyloid A 2	2.01	D
1449326_x_at	Saa2	serum amyloid A 2	2.13	MD
**Transport - upregulated**				
1422008_a_at	Aqp3	aquaporin 3	4.59	I
1450460_at	Aqp3	aquaporin 3	3.10	I
1422007_at	Aqp3	aquaporin 3	3.03	I
1429727_at	Slc16a9	solute carrier family 16 (monocarboxylic acid transporters), member 9	2.60	I
1419758_at	Abcb1a	ATP-binding cassette, sub-family B (MDR/TAP), member 1A	2.48	I
1421848_at	Slc22a5	solute carrier family 22 (organic cation transporter), member 5	2.19	I
1419759_at	Abcb1a	ATP-binding cassette, sub-family B (MDR/TAP), member 1A	2.07	I
**Transport - downregulated**				
1416854_at	Slc34a2	solute carrier family 34 (sodium phosphate), member 2	4.86	D
1418538_at	Kdelr3	KDEL (Lys-Asp-Glu-Leu) endoplasmic reticulum protein retention receptor 3	2.81	D
1417415_at	Slc6a3	solute carrier family 6 (neurotransmitter transporter, dopamine), member 3	2.13	D
**Apoptosis/ cell cycle - upregulated**				
1448700_at	G0s2	G0/G1 switch gene 2	3.03	I
1451392_at	Rbed1	RNA binding motif and ELMO domain 1	2.28	I
1422470_at	Bnip3	BCL2/adenovirus E1B 19kDa-interacting protein 1, NIP3	2.16	I
1455938_x_at	Rad21	RAD21 homolog (S. pombe)	2.08	I
**Apoptosis/ cell cycle - downregulated**				
1452318_a_at	Hspa1b	heat shock protein 1B	2.46	D
1427127_x_at	Hspa1b / Hspa1a	heat shock protein 1B /// heat shock protein 1A	2.39	D
1417040_a_at	Bok	Bcl-2-related ovarian killer protein	2.11	D
1427126_at	Hspa1b	heat shock protein 1B	2.08	D
**Cell adhesion/ homeostasis/ structure - upregulated**				
1456244_x_at	Txnl2	thioredoxin-like 2	2.55	I
1455232_at	Cml2	camello-like 2	2.20	I
1449269_at	F5	coagulation factor V	2.14	I
1452679_at	Tubb2b	RIKEN cDNA 2410129E14 gene	2.08	I
**Cell adhesion/ homeostasis/ structure - downregulated**				
1437258_at	Sprr2a	small proline-rich protein 2A	3.01	D
**Digestion - upregulated**				
1415954_at	Try4 /// Prss1 /// 1810049H19Rik /// LOC436522	trypsinogen 16 /// RIKEN cDNA 1810049H19 gene /// trypsin 4	5.58	I
1428102_at	Cpb1	Carboxypeptidase B1 (tissue)	5.03	I
1435507_x_at	Prss2	protease, serine, 2	4.29	I
1448220_at	Ctrb1	chymotrypsinogen B1	4.26	I
1433573_x_at	Prss2	protease, serine, 2	3.89	I
1417682_a_at	Prss2	protease, serine, 2	3.81	I
1433459_x_at	Prss2	protease, serine, 2	3.73	I

1431763_a_at	Ctrl	chymotrypsin-like	2.99	I
**Immune system - upregulated**				
1418734_at	H1-K1 / H2-Q1 / H2-Q2	histocompatibility 2, K1, K region /// histocompatibility 2, Q region locus 1 /// histocompatibility 2, Q region locus 2	2.13	I
1448235_s_at	Hmgb1 /// 4932431P20Rik	RIKEN cDNA 4932431P20 gene /// high mobility group box 1	2.08	MI
**Immune system - downregulated**				
1425137_a_at	H2-Q10	histocompatibility 2, Q region locus 10	8.22	D
1418787_at	Mbl2	mannose binding lectin, serum (C)	3.18	D
1418652_at	Cxcl9	chemokine (C-X-C motif) ligand 9	2.00	D
**Hemopoiesis - upregulated**				
1422492_at	Cpox	coproporphyrinogen oxidase	2.30	I
**Hemopoiesis - downregulated**				
1420871_at	Gucy1b3	guanylate cyclase 1, soluble, beta 3	2.27	D
1417311_at	Crip2	cysteine rich protein 2	2.07	MD
**Transcription/ translation - upregulated**				
1438487_s_at	Zzz3	zinc finger, ZZ domain containing 3	2.38	I
1438211_s_at	Dbp	D site albumin promoter binding protein	2.28	I
1423142_a_at	Gtpbp4	GTP binding protein 4	2.06	I
**Signal transduction - upregulated**				
1428942_at	Mt2	metallothionein 2	2.77	I
1429206_at	Rhobtb1	Rho-related BTB domain containing 1	2.35	I
**Steroid hormone metabolism - upregulated**				
1426856_at	Hsdl2	hydroxysteroid dehydrogenase like 2	2.60	I
1460232_s_at	Hsd3b2 /// Hsd3b3 /// Hsd3b6	hydroxysteroid dehydrogenase-2, delta<5>-3-beta /// hydroxysteroid dehydrogenase-6, delta<5>-3-beta	2.50	I
1427377_x_at	Hsd3b3	Hydroxysteroid dehydrogenase-3, delta<5>-3-beta	2.00	I
**Miscellaneous/ unknown - upregulated**				
1422434_a_at	2210010C04Rik	RIKEN cDNA 2210010C04 gene	4.79	I
1448894_at	Akr1b8	aldo-keto reductase family 1, member B8	4.26	I
1428005_at	Mosc1	MOCO sulphurase C-terminal domain containing 1	3.86	I
1422435_at	2210010C04Rik	RIKEN cDNA 2210010C04 gene	3.53	I
1417769_at	---	---	2.95	I
1442445_at	2610027H17Rik	RIKEN cDNA 2610027H17 gene	2.81	I
1457619_at	BC015286	cDNA sequence BC015286	2.68	I
1440325_at	---	Adult male medulla oblongata cDNA, RIKEN full-length enriched library, clone:6330531L09 product:unknown EST, full insert sequence	2.66	I
1455454_at	Akr1c19	Similar to 3(20)alpha-hydroxysteroid/dihydrodiol/indanol dehydrogenase	2.55	I
1425470_at	---	---	2.51	I
1424211_at	5730438N18Rik	RIKEN cDNA 5730438N18 gene	2.48	I
1443673_x_at	---	---	2.46	I
1424716_at	0610039N19Rik	RIKEN cDNA 0610039N19 gene	2.45	I
1438786_a_at	2610021A01Rik /// LOC668572	RIKEN cDNA 2610021A01 gene /// similar to zinc finger protein 347	2.39	I
1434424_at	9630055N22Rik /// LOC670389	RIKEN cDNA 9630055N22 gene	2.30	I
1436330_x_at	LOC278757 /// LOC631624	similar to reduced expression 2 /// similar to reduced expression 2	2.28	I
1452565_x_at	LOC641050	hypothetical protein LOC641050	2.28	I
1429527_a_at	Plscr1	phospholipid scramblase 1	2.20	I
1438259_at	---	---	2.16	I
1429822_at	4633401B06Rik	RIKEN cDNA 4633401B06 gene	2.16	I
1448964_at	S100g	S100 calcium binding protein G	2.16	I
1424913_at	2310044G17Rik	RIKEN cDNA 2310044G17 gene	2.13	I
1449345_at	2810027O19Rik	RIKEN cDNA 2810027O19 gene	2.13	I
1431916_at	9030618K22Rik	RIKEN cDNA 9030618K22 gene	2.11	I
AFFX-TransRecMur/ X57349_M_at	Tfrc	transferrin receptor	2.11	I
1437873_at	6030490I01Rik	RIKEN cDNA 6030490I01 gene	2.07	I
1448281_a_at	Ela2	elastase 2	2.04	I
1443773_at	Ylpm1	YLP motif containing 1	2.46	MI
**Miscellaneous/ unknown - downregulated**				

1419591_at	Mlze	melanoma-derived leucine zipper, extra-nuclear factor	4.56	D
1430641_at	9030605I04Rik /// AI987692	RIKEN cDNA 9030605I04 gene /// expressed sequence AI987692	3.97	D
1454883_at	AI987692	expressed sequence AI987692	3.58	D
1425099_a_at	Arntl	aryl hydrocarbon receptor nuclear translocator-like	2.50	D
1440157_at	Scml4	Sex comb on midleg-like 4 (Drosophila)	2.50	D
1422137_at	9030623N16Rik	RIKEN cDNA 9030623N16 gene	2.48	D
1455531_at	A930031D07Rik	RIKEN cDNA A930031D07 gene	2.39	D
1443923_at	Akap13	RIKEN cDNA 9630026M06 gene	2.35	D
1426542_at	Endod1	RIKEN cDNA 2310067E08 gene	2.04	D
1435265_at	---	Adult male cecum cDNA, RIKEN full-length enriched library, clone:9130204C03 product:unclassifiable, full insert sequence	2.00	D
1419098_at	Stom	stomatin	2.00	D
1457296_at	Cilp	cartilage intermediate layer protein, nucleotide pyrophosphohydrolase	2.14	MD

Probesets were selected on the basis of expression in both dietary groups and showing differentially regulation by EPA&DHA (FC ≥ 2, p-value ≤ 0.0027). Grouping per biological process is based on the total number of unique annotated genes (see Table 1), and sorted accordingly. Within each process, probesets are sorted by decreasing FC (increased followed by decreased).

I: increased, D: decreased, M(I or D): medium (increased or decreased) according to Affymetrix MAS5.0 data analysis.
