# Supplementary material for: Induction of lipid oxidation by polyunsaturated fatty acids of marine origin in small intestine of mice fed a high-fat diet
Source: BMC Genomics. 2009 Mar 16;10:110. doi: 10.1186/1471-2164-10-110 (PMC2662879; doi:10.1186/1471-2164-10-110)
Supplement: Additional file 2 — Additional data analysis of gene expression differences. Additional and extended microarray data analysis results are described in more detail. [file 1471-2164-10-110-S2.pdf]

Additional file 2. Additional data analysis of gene expression differences.

This supplemental analysis document describes in more detail the microarray data analyses and its results obtained using the genes expressed in both control and intervention group. It is therefore an additional document, and although the main findings are described in the manuscript and some overlap is insuperable, it will provide more detailed information on the differentially regulated genes. References mentioned here are listed at the end of this document.

Effects of EPA&DHA on small intestinal gene expression; analysis of genes expressed in both control and intervention group.

To investigate the effects of EPA&DHA on gene expression in the intestinal tract, we isolated RNA from scrapings from small intestine of mice following a 4-week dietary intervention. We compared, using whole genome microarray analysis as initial step, the mice fed the control sHF diet, which was rich in ALA and free of EPA or DHA, with the mice fed the isocaloric sHFf-F2 diet, in which 44 % of lipids were replaced by an EPA and DHA concentrate.

In total, 1474 probesets showed a significant change in expression by EPA&DHA compared to control diet ($p<0.0027$, Affymetrix criteria - see methods section-; downregulated probesets with $-9.78 \leq$ fold change (FC) ≤ -1.19 and upregulated probesets with $1.11 \leq FC \leq 6.96$). As dietary intervention at physiological levels is known to generally give small effects on gene expression changes ([1, 2], own unpublished observations), we performed initial pathway analyses using an absolute $FC \geq 1.5$ for all significantly regulated genes, which resulted in a total of 621 probesets (420 increased, 201 decreased). Metabolism,

and especially lipid metabolism, was identified as the major regulated process. A similar approach using a more stringent condition, FC ≥ 2.0, resulted in 155 probesets (Supplementary Table 1), of which 130 were annotated genes. Increased expression was observed for 99 annotated probesets (73 unique genes), while 31 annotated probesets (27 unique genes) showed decreased expression (Table 1). Pathway analysis using those 130 probesets resulted in a similar list of differentially regulated pathways when we compared these results with the list obtained using FC ≥ 1.5 (data not shown). Again, metabolism was highly regulated (49% of differentially expressed genes) and involved changes in fatty acid uptake, fatty acid oxidation and cholesterol biosynthesis, amongst others. Of note, most, if not all, pathways selected were linked together by the energy molecule acetyl-CoA. Based on the sufficiently large group of genes with FC ≥ 2.0, we focussed on those 100 unique, differentially expressed genes (see Table 1).

Detailed inspection of the expression data (see Additional file 1; see also for full names of the genes) revealed that EPA&DHA induced expression of the genes mediating fatty acid uptake from the lumen into intestinal tissue, namely of *Cd36* (FC=2.3) and *Scarb1* (FC=2.2). However, expression of the class of fatty acid binding proteins (*Fabp1,2,4-6*), including intestinal-specific *Fabp2*, was not changed. Remarkably, small intestine of the EPA&DHA mice showed also increased expression of the genes of fatty acid β-oxidation (both in peroxisomes and mitochondria) and fatty acid ω-oxidation, the latter is indicated by the increased expression of *Cyp4a10* (FC=5.6).

In more detail, the genes involved in both branched chain and straight chain fatty acid β-oxidation with a more than two fold increase due to the intake of EPA&DHA were *Acox2* (FC=2.9) and *Hsd17b4* (FC=2.0), while *Scp2* (FC=1.6), *Crot* (FC=1.7), and *Scl27a2* (FC=1.4) were increased to a lesser extent. In addition, branched chain specific gene *Aldh3a2* (FC=1.8), and straight chain specific genes *Acaa1a* (FC=1.7), *Acaa1b* (FC=2.2), *Acox1* (FC=1.4), and

Ehahdh (FC=1.6) were increased as well. Furthermore, peroxisomal *Acot8* (FC=1.3) and *Acot4* (FC=1.8) also increased. Induction of the peroxisomal biogenesis factor gene *Pex11a* (FC=2.2) might imply an increase in the number of peroxisomes. Finally, a peroxisome membrane protein with unknown function (*Pxmp4*) was upregulated (FC=2.2).

In mitochondria, β-oxidation downstream of peroxisomal branched chain oxidation was upregulated by EPA&DHA, as suggested by the increased expression of the genes *CPT1a* (FC=2.9), *Hadhb* (FC=2.2), *Acadl* (FC=1.6), *Acaa2* (FC=2.0), *Cpt2* (FC=1.4), and *Hadha* (FC=1.4). Activity of carnitine palmitoyltransferase 1 (encoded by *Cpt1a*) is rate-limiting in mitochondrial fatty acid uptake for β-oxidation. The above genes are also involved in long chain β-oxidation. The gene encoding 3-hydroxy-3-methyglutaryl-CoA synthase *(Hmgcs2)* was also very strongly upregulated (FC=4.6), in accordance with the fact that in liver this enzyme is the rate limiting enzyme in the synthesis of ketone bodies from the acetyl-CoA generated by fatty acid β-oxidation. The gene for mitochondrial *Acot2* (FC=2.0) increased as well, suggesting increased transport of fatty acids across the mitochondrial membrane. Expression of cytosolic *Acot1* (FC=5.1) and *Acot12* (FC=1.6) was also increased. Importantly, also expression of *Pdk4* was increased by EPA&DHA (FC=3.0), which strongly suggested a switch from glycolysis to fatty acid oxidation [3]. Many of the aforementioned genes are targets of peroxisome proliferator activated receptor (*PPAR)* alpha [4], which itself was also upregulated by EPA&DHA (FC=2.0).

Of the 21 genes included in the cholesterol biosynthesis pathway from acetyl-CoA to cholesterol, five are inhibited in their expression by EPA&DHA (FC≤-2.0), including the rate-limiting enzyme squalene epoxidase (*Sqle*, FC=-2.1). Additionally, four genes showed FC ≤ -1.5, while 6 and 4 genes are non-significantly inhibited and induced, respectively. The remaining two genes in this pathway (*Idi2* and *Mvk*) are not expressed in both diet groups. Clearly, such a cooperative inhibition of the majority of the genes in this pathway suggests an

orchestrated function within the small intestine. However, the main transcription factor regulating this pathway, *Srebf1* (the mouse counterpart of human SREBP), did not show differential expression. Interestingly, elastase 3B (FC=8.7) and *ScarB1* (FC=2.2), two putative transporters of cholesterol, were upregulated, as was the known cholesterol transporter *Abca1* (FC=1.4). Regulation by n-3 PUFAs was likely given the fact that we could not detect a difference in the cholesterol content between the two diets (data not shown), but the mechanisms involved remain unexplained. Finally, downstream steroid hormone biosynthesis was upregulated as shown by a few family members of *Hsd3b* (*Hsd3b2*: FC=2.3; *Hsd3b3* FC=2.0) and *Hsd17b* (*Hsd17b4* two probesets: FC=1.6 and 2.0; *Hsd17b13* FC=2.4).

References

1. Afman L, Muller M: **Nutrigenomics: from molecular nutrition to prevention of disease**. *J Am Diet Assoc* 2006, **106**(4):569-576.
2. Pagmantidis V, Meplan C, van Schothorst EM, Keijer J, Hesketh JE: **Supplementation of healthy volunteers with nutritionally relevant amounts of selenium increases the expression of lymphocyte protein biosynthesis genes**. *Am J Clin Nutr* 2008, **87**(1):181-189.
3. Sugden MC: **PDK4: A factor in fatness?** *Obes Res* 2003, **11**(2):167-169.
4. Mandard S, Muller M, Kersten S: **Peroxisome proliferator-activated receptor alpha target genes**. *Cell Mol Life Sci* 2004, **61**(4):393-416.
