# Supplementary material for: Induction of lipid oxidation by polyunsaturated fatty acids of marine origin in small intestine of mice fed a high-fat diet
Source: BMC Genomics. 2009 Mar 16;10:110. doi: 10.1186/1471-2164-10-110 (PMC2662879; doi:10.1186/1471-2164-10-110)
Supplement: Additional file 3 — Transcription factors enriched for involvement with differentially expressed genes. The data provided represent identified transcription factors on the basis of an enriched presence of TFBS in the promoter region of the set of significantly differentially regulated genes, combined with data from scientific publications. [file 1471-2164-10-110-S3.pdf]

Additional file 3. Transcription factors enriched for involvement with differentially expressed genes.

**Transcription Factor Symbol**	**Name**	**Number of linked input genes (%)**
PPARalpha	peroxisome proliferator activated receptor alpha	27 (54%)
NF-κB	nuclear factor kappa beta	20 (40%)
Stat3	signal transducer and activator of transcription 3	14 (28%)
Sp1	trans-acting transcription factor 1	10 (20%)
Ahr	aryl-hydrocarbon receptor	7 (14%)
Arnt1	aryl hydrocarbon receptor nuclear translocator	4 (8%)
Dbp	D site albumin promoter binding protein	2 (4%)
