# Supplementary material for: Induction of lipid oxidation by polyunsaturated fatty acids of marine origin in small intestine of mice fed a high-fat diet
Source: BMC Genomics. 2009 Mar 16;10:110. doi: 10.1186/1471-2164-10-110 (PMC2662879; doi:10.1186/1471-2164-10-110)
Supplement: Additional file 4 — Differentially regulated probesets expressed in only one dietary group. The data provided represent all differentially expressed probesets of the microarrays which are unique for either one of the dietary groups. [file 1471-2164-10-110-S4.pdf]

Additional file 4. Differentially regulated probesets expressed in only one dietary group.

Probeset ID	Gene Symbol	Gene Title	FC	I/D
**metabolism**				
1424943_at	Cyp4a10	cytochrome P450, family 4, subfamily a, polypeptide 10	21.26	I
1427052_at	Acacb	acetyl-Coenzyme A carboxylase beta	6.87	I
1422925_s_at	Acot3	acyl-CoA thioesterase 3	5.74	I
1445980_at	Aldh1a1	Aldehyde dehydrogenase family 1, subfamily A1	2.01	I
1441413_at	Cat	Catalase	2.39	I
1435371_x_at	Ces3	carboxylesterase 3	3.10	I
1428062_at	Cpa1	carboxypeptidase A1	3.34	I
1454623_at	Cpa2	carboxypeptidase A2, pancreatic	16.22	I
1440134_at	Cyp4a10	cytochrome P450, family 4, subfamily a, polypeptide 10	4.32	I
1425627_x_at	Gstm1 /// LOC433943	glutathione S-transferase, mu 1	2.27	I
1425127_at	Hsd3b2	hydroxysteroid dehydrogenase-2, delta<5>-3-beta	2.30	I
1453836_a_at	Mgll	monoglyceride lipase	2.55	I
1419000_at	Cpxm2	carboxypeptidase X 2 (M14 family)	8.00	D
1434862_at	Fut2	fucosyltransferase 2	3.10	D
1434499_a_at	Ldhb	lactate dehydrogenase B	14.93	D
1452940_x_at	Pitpnc1	phosphatidylinositol transfer protein, cytoplasmic 1	2.00	MD
**transcription/translation/splicing**				
1437262_x_at	Bcas2	breast carcinoma amplified sequence 2	5.58	I
1459828_at	Srrm1	Serine/arginine repetitive matrix 1	2.03	I
1441876_x_at	Zfp93	Zinc finger protein 93	9.19	MI
1459804_at	Crebbp	CREB binding protein	15.24	D
1420573_at	Hoxd1	homeo box D1	6.96	D
1431134_at	Ing5	inhibitor of growth family, member 5	2.19	D
1426498_at	Jarid1c	jumonji, AT rich interactive domain 1C (Rbp2 like)	5.39	D
1427400_at	Lbx1	ladybird homeobox homolog 1 (Drosophila)	3.10	D
1446389_at	Nrip1	Nuclear receptor interacting protein 1	2.06	D
**cell adhesion/ proliferation/ differentiation/ structure**				
1447077_at	Add3	Adducin 3 (gamma)	19.16	D
1416077_at	Adm	adrenomedullin	2.50	D
1426283_at	Hnt	neurotrimin	2.81	D
1448201_at	Sfrp2	secreted frizzled-related protein 2	6.41	D
1417136_s_at	Srpk2	serine/arginine-rich protein specific kinase 2	2.31	D
1422047_at	Cdh5	cadherin 5	5.03	MD
1438410_at	Prtg	protogenin homolog (Gallus gallus)	4.53	MD
**signal transduction**				
1417331_a_at	Arl6	ADP-ribosylation factor-like 6	4.38	D
1424376_at	Cdc42ep1	CDC42 effector protein (Rho GTPase binding) 1	2.53	D
1425856_at	Cib1	Calcium and integrin binding 1 (calmyrin)	2.87	D
1456613_at	Eif4ebp2	eukaryotic translation initiation factor 4E binding protein 2	2.41	D
1416266_at	Pdyn	prodynorphin	3.34	D
1446918_at	Lphn3	Latrophilin 3	5.74	MD
1424963_at	Rp1h	retinitis pigmentosa 1 homolog (human)	4.35	MD
**transport**				
1436770_x_at	Psma1	proteasome (prosome, macropain) subunit, alpha type 1	25.81	I
1439843_at	Camk4	calcium/calmodulin-dependent protein kinase IV	2.75	D
1445434_at	Snupn	Snurportin 1	4.63	D

1447856_x_at	Slc25a25	solute carrier family 25 (mitochondrial carrier, phosphate carrier), member 25	2.73	D
1443676_at	Slc4a4	Solute carrier family 4 (anion exchanger), member 4	3.58	D
1418030_at	Slco3a1	solute carrier organic anion transporter family, member 3a1	6.45	D
**protein folding/modification**				
1428474_at	Ppp3cb	protein phosphatase 3, catalytic subunit, beta isoform	2.10	I
1423553_at	Dnajb3	DnaJ (Hsp40) homolog, subfamily B, member 3	2.48	D
1428640_at	Hsf2bp	heat shock transcription factor 2 binding protein	2.13	D
1438253_at	Ssh1	slingshot homolog 1 (Drosophila)	8.28	D
1416504_at	Ulk1	Unc-51 like kinase 1 (C. elegans)	3.07	D
**cell cycle**				
1456744_x_at	Flcn	folliculin	8.63	I
1431605_at	Rb1cc1	RB1-inducible coiled-coil 1	2.00	MD
**growth factor**				
1457123_at	Nrg4	neuregulin 4	3.12	I
1418376_at	Fgf15	fibroblast growth factor 15	7.11	D
**miscellaneous/unknown**				
1435132_at	Disp1	dispatched homolog 1 (Drosophila)	5.82	I
1448600_s_at	Vav3	vav 3 oncogene	4.86	I
1443043_at	Otop2	otopetrin 2	2.07	I
1416929_at	Rbm12	RNA binding motif protein 12	2.43	I
1417122_at	Vav3	vav 3 oncogene	3.20	I
1443179_at	Csmd1	CUB and Sushi multiple domains 1	13.27	MI
1439144_at	Cwf19l1	CWF19-like 1, cell cycle control (S. pombe)	2.57	D
1458012_at	Dach2	dachshund 2 (Drosophila)	48.17	D
1451853_at	Fcmd	Fukuyama type congenital muscular dystrophy homolog (human)	2.39	D
1419196_at	Hamp1	hepcidin antimicrobial peptide 1	3.97	D
1436743_at	Igsf4d	immunoglobulin superfamily, member 4	42.81	D
1438445_at	Lrrc8e	leucine rich repeat containing 8 family, member E	12.38	D
1449989_at	Mcpt2	mast cell protease 2	2.73	D
1457113_at	Nasp /// D4Ertd767e	nuclear autoantigenic sperm protein (histone-binding)	4.66	D
1431896_at	Six6os1	Six6 opposite strand transcript 1	24.25	D
1434429_at	Syt16	synaptotagmin XVI	2.25	D
1442074_at	Clec2g	C-type lectin domain family 2, member g	2.46	MD
1446777_at	Spp2	Secreted phosphoprotein 2	7.41	MD

Probesets were selected on the basis of expression in only one dietary group and showing differential regulation by EPA&DHA (absolute fold change (FC) ≥ 2, p-value ≤ 0.0027). Grouping per biological process is based on the total number of unique annotated genes (see Table 2), and sorted accordingly. Within each process, probesets are sorted by decreasing FC (increased followed by decreased). I: increased, D: decreased, M (I or D): medium (increased or decreased) according to Affymetrix MAS5.0 data analysis.
