# Supplementary material for: Induction of lipid oxidation by polyunsaturated fatty acids of marine origin in small intestine of mice fed a high-fat diet
Source: BMC Genomics. 2009 Mar 16;10:110. doi: 10.1186/1471-2164-10-110 (PMC2662879; doi:10.1186/1471-2164-10-110)
Supplement: Additional file 5 — Real-time quantitative PCR: genes and primers. Gene Symbols, Names, Annotation (RefSeq), and primer sequences for genes analyzed using qRT-PCR are shown. [file 1471-2164-10-110-S5.pdf]

Additional file 5. Real-time quantitative RT-PCR: genes and primers

gene symbol	gene name	Accession number (RefSeq)	Forward primer (5' – 3')	Reverse primer (5' – 3')
Acaa1a	acetyl-Coenzyme A acyltransferase 1A, 3-ketoacyl-CoA thiolase A	NM_130864	CGGGAGCAAGGCAGGTTGTC	GCTGAGTGTCCCACGCAGAC
Acaa1b	acetyl-Coenzyme A acyltransferase 1B, 3-ketoacyl-CoA thiolase B	NM_146230	CGGGAGCAAGGCAGGTTGTC	GCTGAGTGTCCCACGCAGAC
Acacb	acetyl-Coenzyme A carboxylase beta	NM_133904	ACTGTGGAGTATCTCGTTAACCTTCTG	GGACTGTCGGGCTACCTTGAG
Acox1	acyl-Coenzyme A oxidase 1, palmitoyl	NM_015729	GCAGATAAACTCCCCAAGATTCAAGAC	TAAAGTCAAAGGCATCCACCAAAGC
Acox2	acyl-Coenzyme A oxidase 2, branched chain	NM_053115	CGGAAGGACGCCATCTTGTTAAC	TGGTCCTGAATCCTTTGGCTTTTC
Acox3	acyl-Coenzyme A oxidase 3, pristanoyl	NM_030721	TCTACCGAGGTGGCTACATTTCTG	GCACGCTCTAGCACACCATTC
Cpt1a	carnitine palmitoyltransferase 1a, liver	NM_013495	GCTGATGACGGCTATGGTGTTTC	TTGTATCACTAGAGTCCATTTTCCTTCC
Cpt2	carnitine palmitoyltransferase 2	NM_009949	GCTCCGAGGCATTTGTCAGG	TTGTGGTTTATCCGCTGGTATGC
Ela2	elastase 2	NM_007919	GGTGGGGAAGCTCTGTGAAGTC	GACCCTGGTGAAGACGGATGG
Ela3b	elastase 3B, pancreatic	NM_026419	GCAACGGTGACTCTGGAGGAC	GCCAGCCGAACCTTGGATCTAG
H2Q10	histocompatibility 2, Q region locus 10	NM_010391	GAGAAGGAGAAACACAGGTAGAAAAGG	CCACAGGAACACAGAGAACATCAG
Hmgcs1	3-hydroxy-3-methylglutaryl-Coenzyme A synthase 1	NM_145942	GCCCCTTCACAAATGACCACAG	CATTCCTCCAACCGTTTCCATACC
Hmgcs2	3-hydroxy-3-methylglutaryl-Coenzyme A synthase 2	NM_008256	CTACCTGCGGGCCTTGGATC	GCCCTTGTATAAGTTGTTCTGTTTGTC
Hsd3b	3-beta-hydroxysteroid dehydrogenase (family member 2, 3 and 6)	NM_001012306	CAGGTGCTGTTGCCTTCTTTTGA	AGGCTATACATGCTYTGAATGSGGAG
Mod1	malic enzyme, supernatant	NM_008615	GAGGTCATATCTCAGCAAGTGTCAG	AATCAGGTAGGATCTGGTCATAATTAGTG
Pck1	phosphoenolpyruvate carboxykinase 1, cytosolic	NM_011044	GTTTGTAGGAGCAGCCATGAGATC	CCAGAGGAACTTGCCATCTTTGTC
Pdk4	pyruvate dehydrogenase kinase, isoenzyme 4	NM_013743	CTTACAATCAAGATTTCTGACCGAGGAG	ATAATGTTTGAAGGCTGACTTGTTAAAGAC
Sqle	squalene epoxidase	NM_009270	GCTTTCTGTATTTTAAACTTGGTGGAGAG	AGTGGAAATAGGATAGAACACGCTTTG
Canx	calnexin (reference gene)	NM_007597	GCAGCGACCTATGATTGACAACC	GCTCCAAACCAATAGCACTGAAAGG
Hprt1	hypoxanthine phosphoribosyltransferase (reference gene)	NM_013556	TGACACTGGTAAAACAATGCAAACTTTG	GAGGTCCTTTTCACCAGCAAGCT
